# Energy-dense nutrient-poor snacks and risk of non-alcoholic fattyliver disease: a case–control study in Iran

**DOI:** 10.1186/s13104-020-05063-9

**Published:** 2020-04-16

**Authors:** Zahra Yari, Makan Cheraghpour, Vahideh Aghamohammadi, Meysam Alipour, Nila Ghanei, Azita Hekmatdoost

**Affiliations:** 1 Student Research Committee, Department of Clinical Nutrition and Dietetics, Faculty of Nutrition and Food Technology, National Nutrition and Food Technology Research Institute, Shahid Beheshti University of Medical Sciences, Tehran, Iran; 2 Cancer Research Center, Ahvaz Jundishapur University of Medical Sciences, Ahvaz, Iran; 3 Department of Nutrition, Khalkhal University of Medical Sciences, Khalkhal, Iran; 4 Alimentary Tract Research Center, Imam khomeini Hospital Clinical Research Development Unit, Ahvaz Jundishapur University of Medical Sciences, Ahvaz, Iran; 5 Department of Drug Discovery and Development, Harrison School of Pharmacy, Auburn University, Auburn, USA; 6 Department of Clinical Nutrition and Dietetics, Faculty of Nutrition Sciences and Food Technology, National Nutrition and Food Technology Research Institute, Shahid Beheshti University of Medical Science, Tehran, Iran

**Keywords:** Non-alcoholic fatty liver disease, Energy-dense snack, Nutrient-poor snack

## Abstract

**Objective:**

The purpose of the present study was to determine the association between energy-dense nutrient-poor snacks intake and the risk of non-alcoholic fatty liver disease (NAFLD) in Iranian adults. For this purpose, a total of 143 cases with a newly confirmed diagnosis of NAFLD and 471 controls free of the disease were studied. Dietary intake was assessed using a food frequency questionnaire.

**Results:**

The percentage of calories from total energy-dense nutrient-poor snacks was 6.08% and 5.04%, in patients and controls, respectively (P = 0.036). Compared with subjects in the lowest quartile of total energy-dense nutrient-poor snacks intake, the risk of NAFLD for those in the top quartile of consumption increased by about two times, in both crude (OR: 1.94; 95% CIs 1.16–3.26; P for trend = 0.015) and adjusted (OR: 2.27; 95%CIs 1.19–4.31; P for trend = 0.001) models. The relative odds of NAFLD increased significantly in the fourth quartile of dietary cake and biscuit (OR: 1.21, P for trend = 0.037) and soft drinks (OR: 1.64, P for trend = 0.005) intake compared with the lowest corresponding quartiles, after adjustment for age, sex, body mass index, physical activity, alcohol, energy intake. Our results indicate that there might be a moderate positive association between energy-dense nutrient-poor snacks intake and risk of NAFLD.

## Introduction

Non-alcoholic fatty liver disease (NAFLD) is defined as an accumulation of more than 5% fat in the hepatocytes [1]. The pooled global prevalence of NAFLD is estimated to be 24.4% in the general population [2]. NAFLD can lead to severe liver pathologies, including fibrosis, cirrhosis and hepatocellular carcinoma. Evidences from literature demonstrate that the effects of NAFLD develop beyond the liver and are associated with a wide range of chronic diseases, most especially cardiovascular disease (CVD) and diabetes mellitus type 2 (T2DM) [3]. Weight gain, dietary changes with shift to a westernized diet, higher consumption of industrial and processed foods and sedentary lifestyle may contribute to the increasing trend of NAFLD; therefore at present, weight loss through lifestyle modification is the first-line approach for the management of NAFLD [4].

Snacking patterns may play a role on the occurrence of metabolic abnormalities with effects on energy and nutrient intakes.

In general, two groups of food fall under the category of snacks. One is energy-dense nutrient-poor (such as potato chips, chocolates, candies, biscuits, cakes, sweets and sugar sweetened beverages) and the other one is low-energy high-nutrient (such as vegetables, fruits, and skim milk) [5]. Energy-dense nutrient-poor snacks are rich sources of simple sugars, fructose, sodium, saturated and trans fatty acids [6]. These unhealthy snacks are one of the major concerns regarding NAFLD development [7]. Excessive added sugar in snacks through increasing calorie intake may lead to impaired glucose homeostasis, induction of lipogenesis and β-cell dysfunction, all of which are risk factors for metabolic syndrome that are closely linked to NAFLD [8]. However, the association of total daily calorie intake from energy-dense nutrient-poor snacks with risk of NAFLD has not yet been well determined. The aim of the current case–control study was to determine the association between consumption of total and various energy-dense nutrient-poor snacks and NAFLD risk in Iranian adult population.

## Main text

### Materials and methods

Study protocol was described previously in details [4, 9]. Briefly, 143 patients with NAFLD and 471 controls were included in the present case–control study. The cases were patients with NAFLD diagnosed by a gastroenterologist for presence of hepatic steatosis in ultrasound exam during previous month who were referred to a tertiary hepatology clinic to be examined by Fibroscan. Controlled attenuation parameter (CAP) score of more than 263 and fibrosis score > 7 in the Fibroscan result were two criteria for NAFLD diagnosis. Controls were randomly selected age-matched subjects from the same clinic among patients who had no evidence of hepatic steatosis on the ultrasound exam. All participants were recruited using convenience sampling method based on inclusion criteria and written consent was obtained. The local Ethics Review Committee approved the study protocol.

At baseline, participants were asked about their age, employment, marital status, education, smoking, past medical history, current use of medications and other factors during a 45-min in-person interview. Physical activity level as metabolic equivalent hours per week (METs h/wk) was evaluated using questionnaire. Also, a valid and reliable 168-item semi quantitative food frequency questionnaire (FFQ) was used to assess dietary intakes [10]. The data from the FFQ was used to determine foods consumption frequency on a daily, weekly or monthly basis during the past year with standard portion sizes, as commonly consumed by Iranians. Data obtained from FFQ were converted to gram intake per day for each food item in order to assess the nutrient and total energy intakes using the Nutritionist 4 software (First Data bank) [11].

Energy-dense nutrient-poor snacks, in this study, were divided into four categories as follow: biscuits and cakes (biscuits, crackers, cakes, cookies and traditional Iranian confectioneries such as gaz, sohan, noghl, halva, Yazdi cakes), candies and chocolates, salty snacks (potato chips, puff snacks) and soft drinks. Fruits, dairy products, and cereals with low or medium energy density and high nutritional value were not considered as snacks. As a whole, these four groups formed total snacks. All these practices were done by a trained dietitian.

### Statistical analysis

All statistical analyses were carried out using SPSS (Version 21.0; Chicago, IL, USA), and P-values at < 0.05 were considered significant. Comparison of baseline characteristics and dietary intakes between two study groups were performed using Student t-test for continuous variables and Chi-square for categorical variables. To evaluate associations between energy-dense nutrient-poor snacks and NAFLD risk, the study participants were divided into 4 categories on the basis of quartiles of total snacks intake and the lowest quartile was set as the reference category. ANOVA test was used to compare the variables between quartiles and P for trend was calculated using linear regression. Odds ratios (ORs) and 95% confidence intervals (CIs) were calculated using multiple logistic regression analysis. Analyses were adjusted for age, sex, body mass index (kg/m^2^), physical activity (MET-h/wk), alcohol and energy intake (kcal/day). Also, to perform the linear trend tests, quartile-specific medians were assigned to each quartile.

### Results

Cases and controls showed no statistical difference in age, gender, and alcohol intake distribution. Cases had significantly higher body mass index (BMI) (31.9 vs. 27.2 kg/m^2^; P < 0.001) and lower physical activity (32.2 vs. 34.2 MET-h/wk; P < 0.001) compared with controls. Energy, carbohydrate, dietary fiber and simple sugars intake were not significantly different between the two study groups.

Basic characteristics and dietary intakes of the study participants across quartiles of total energy-dense nutrient-poor snacks are presented in Table 1. Compared to those in the first quartile of total energy-dense nutrient-poor snacks, subjects in the fourth quartile were younger, consumed more alcohol and had less physical activity. Also, the intake of energy, simple sugar and snacks-derived energy were significantly higher in those in the top quartile compared to the bottom quartile.

**Table 1:** Basic characteristics and dietary intakes of study participants by quartiles of total energy-dense nutrient-poor snacks

	Quartiles of total energy-dense nutrient-poor snacks				*P* trend
	Quartile 1 (n = 155)	Quartile 2 (n = 153)	Quartile 3 (n = 153)	Quartile 4 (n = 153)	
Cases (n)	31	28	34	50	0.013
Age (year)	41.68 ± 9.57	39.99 ± 9.46	38.18 ± 9.35	35.84 ± 9.64	< 0.001
Male/female (%)	46/54	51/49	42/58	57/43	0.042
Weight (kg)	78.66 ± 16.17	76.47 ± 14.66	78.60 ± 15.8	81.4 ± 16.47	0.071
BMI (kg/m^2^)	28.34 ± 5.14	27.8 ± 4.64	28.59 ± 5.68	28.61 ± 6.44	0.424
Physical activity (MET)	34.01 ± 3.21	34.18 ± 3.17	33.93 ± 3	33.07 ± 3.12	0.007
Alcohol (n)*	6	22	13	21	0.006
Dietary factors					
Total energy intake (kcal)	2556.33 ± 764.25	2732.64 ± 823.97	2895.42 ± 716.03	3387.51 ± 974.86	< 0.001
Energy-dense nutrient-poor snack (% energy)	1.56 ± 0.93	3.69 ± 1.62	6.14 ± 2.67	9.74 ± 5.13	< 0.001
Carbohydrate (% energy)	57.77 ± 6.84	57.89 ± 11.59	56.89 ± 7.17	55.94 ± 5.65	0.027
Simple sugar (g)	132.66 ± 80.9	132.61 ± 52.89	136.31 ± 48.68	159.28 ± 51.56	< 0.001
Dietary fiber (g/1000 kcal)	16.06 ± 4.76	15.83 ± 4.88	15.27 ± 5.25	13.11 ± 4.08	< 0.001
Total fat (% energy)	31.67 ± 6.37	32.56 ± 5.48	33.38 ± 5.62	33.48 ± 4.56	0.002
SFA (% energy)	7.96 ± 3.19	8.77 ± 3.41	8.88 ± 3.6	8.54 ± 3.39	0.126
MUFA (% energy)	10.04 ± 2.16	10.56 ± 2.04	10.73 ± 1.93	10.82 ± 1.8	< 0.001
PUFA (% energy)	12.1 ± 5.53	12.52 ± 6.76	12.65 ± 6.23	11.69 ± 5.6	0.618
Protein (% energy)	14.7 ± 2.82	14.57 ± 2.6	14.51 ± 2.53	14.62 ± 2.64	0.763

*BMI* body mass index, *MET* metabolic equivalent task, *SFA* saturated fatty acid, *MUFA* mono-unsaturated fatty acid, *PUFA* poly-unsaturated fatty acid

^a^Data are presented as mean ± SEM or number

^b^Linear regression

* Numbers indicate the number of people who consumed alcohol. This amount of alcohol consumption in men was less than 20 g/day and in women was less than 10 g/day

ORs and 95% CIs for the risk of NAFLD according to quartiles of snack consumption are indicated in Table 2. When analysis was carried out with snack consumption expressed as quartiles, subjects in fourth quartile were at 1.94 times higher risk of having NAFLD compared to those in the first quartile (OR_quartile_ 4 vs. 1 = 1.94, 95% CI 1.19–4.31; P_trend_ < 0.001). Calculating the risk of NAFLD across snacks consumption quartiles adjusting for age, sex, BMI (kg/m^2^), physical activity (MET-h/wk), alcohol, and energy intake (kcal/day), amplified the observed association (OR_quartile_ 4 vs. 1 = 2.27, 95% CI 1.19–4.31; P trend = 0.001). This association was also present for some of the snack subgroups, including biscuits, cakes and soft drinks in an adjusted model.

**Table 2:** Odds and 95% confidence interval for occurrence of the NAFLD in each quartile categories of snack consumption

	Quartiles of total energy-dense nutrient-poor snacks				*P* trend^a^
	Q1 (n = 155)	Q2 (n = 153)	Q3 (n = 153)	Q4 (n = 153)	
Biscuits and cakes					
Model 1	Ref	0.53 (0.31–0.93)	0.73 (0.43–1.23)	0.97 (0.58–1.60)	0.213
Model 2	Ref	0.56 (0.31–1.04)	0.74 (0.40–1.37)	1.21 (0.65–2.26)	0.037
Candies and chocolates					
Model 1	Ref	0.47 (0.28–0.82)	0.51 (0.30–0.86)	0.96 (0.51–1.40)	0.458
Model 2	Ref	0.58 (0.316–1.05)	0.60 (0.31–1.15)	0.92 (0.85–0.99)	0.169
Salty snacks					
Model 1	Ref	0.65 (0.39–1.11)	0.79 (0.47–1.34)	0.87 (0.52–1.46)	0.507
Model 2	Ref	0.67 (0.37–1.29)	0.72 (0.36–1.37)	0.85 (0.46–1.54)	0.424
Soft drinks					
Model 1	Ref	0.28 (0.15–0.56)	1.23 (0.81–2.16)	1.38 (0.82–2.32)	0.087
Model 2	Ref	0.33 (0.16–0.68)	1.24 (0.67–2.30)	1.64 (0.82–2.65)	0.005
Total snacks					
Model 1	Ref	0.89 (0.51–1.58)	1.14 (0.66–1.98)	1.94 (1.16–3.26)	0.015
Model 2	Ref	0.92 (0.48–1.77)	1.18 (0.63–2.19)	2.27 (1.19–4.31)	0.001

Model 1: Crude

Model 2: Adjustment for age, sex, body mass index (kg/m^2^), physical activity (MET-h/wk), alcohol, energy intake (kcal/day)

^a^Based on multiple logistic regression model

### Discussion

In this case–control study, a positive association was found between a higher consumption of energy-dense nutrient-poor snacks and NAFLD risk among Iranian adult population. The odds of NAFLD increased more than twice in participants who were in the highest quartile of snacks consumption. We also observed that higher intake of cake and biscuits and soft drinks were related to increased risk of NAFLD.

A number of dietary factors have been implicated in the pathogenesis of NAFLD with a recent focus on dietary carbohydrates, sugar-sweetened beverages and the monosaccharide fructose in particular [12]. Increasing consumption of energy-dense nutrient-poor snacks might be due to several factors, including inappropriate eating habits, poor nutrition knowledge, media advertising and easy access to them; most of these are attributed to changes in dietary habits in keeping with the shift from the traditional to the western lifestyle [13]. Unhealthy snacks, on the other hand, usually substitute healthy snacks, including fruits and dairy, which are a part of low-energy high-nutrient diet [14].

Simple sugars as a substrate for lipogenesis can increase hepatic fat content, which may lead to inflammatory cascade and eventually fibrosis. Many sweet snacks contain fructose syrup, which has been shown to be associated with NAFLD. Since there is no negative feedback in the pathway of lipogenesis from fructose, all extra-absorbed fructose can be converted to triglyceride in hepatocytes [9, 12, 14–17]. Also, according to a cross-sectional study in the Iranian adult population, consumption of simple sugars was associated with an increased risk of hypertriglyceridemia (OR: 1.76; 95% Cis 1.01–3.07) that is directly related to NAFLD [18].

Another study found that high intakes of sweets and desserts, soft drinks and potato chips in the western dietary pattern were associated with a 26% increased risk of insulin resistance [19], which has a key role in the pathogenesis of NAFLD. Fatty liver is considered as a hepatic manifestation of metabolic syndrome. Results of Tehran Lipid and Glucose Study showed that the risk of metabolic syndrome in the highest quartile of snacks was 50% higher than the lowest one after 3 years follow-up [20].

We did not find a significant association between consumption of salty snacks and NAFLD. Although high sodium intake is associated with obesity and hypertension, few studies have investigated the relationship between sodium intake and NAFLD. High Sodium intake is associated with incidence of NAFLD and advanced liver fibrosis in young and asymptomatic adults, which might be partly related to adiposity. The correlation between high salt intake and NAFLD could be explained by dysregulation of the rennin-angiotensin system, which has a key role in development of hepatic inflammation and fibrosis and activation of mineralocorticoid receptors that induce free radical production and oxidative stress [21, 22]. In addition, high salt intake is commonly the result of consumption of high energy foods with a high salt content, such as cheese and chips, which leads to increase of total energy intake and consequently increase in body fat [23]. Relatively large sample size of cases and controls and high rate of participation are of advantages of this research. Moreover, current study is the first research evaluating the association between intake of energy-dense nutrient-poor snacks and risk of NAFLD.

### Conclusions

In summary, the results from the current study show that there might be a moderate positive association between sweet energy-dense nutrient-poor snacks intake and risk of NAFLD.

## Limitation

We used a valid and reliable FFQ for assessment of dietary intakes; however, measurement error, and recall bias are inevitable errors. One limitation of this study is that it is performed in relatively small cohort. Observed trends warrant investigation in a larger group of subjects.

## Data Availability

All data generated or analyzed during this study are included in this published article.

## Abbreviations

BMI: Body mass index
CAP: Controlled attenuation parameter
CIs: Confidence intervals
CVD: Cardiovascular disease
FFQ: Food frequency questionnaire
METs h/wk: Metabolic equivalent hours per week
NAFLD: Non-alcoholic fatty liver disease
ORs: Odds ratios
T2DM: Diabetes mellitus type 2
