# Correction to: Energy-dense nutrient-poor snacks and risk of non-alcoholic fattyliver disease: a case–control study in Iran

**DOI:** 10.1186/s13104-020-05306-9

**Published:** 2020-09-29

**Authors:** Zahra Yari, Makan Cheraghpour, Vahideh Aghamohammadi, Meysam Alipour, Nila Ghanei, Azita Hekmatdoost

**Affiliations:** 1 Student Research Committee, Department of Clinical Nutrition and Dietetics, Faculty of Nutrition and Food Technology, National Nutrition and Food Technology Research Institute, Shahid Beheshti University of Medical Sciences, Tehran, Iran; 2 Cancer Research Center, Ahvaz Jundishapur University of Medical Sciences, Ahvaz, Iran; 3 Department of Nutrition, Khalkhal University of Medical Sciences, Khalkhal, Iran; 4 Alimentary Tract Research Center, Imam Khomeini Hospital Clinical Research Development Unit, Ahvaz Jundishapur University of Medical Sciences, Ahvaz, Iran; 5 Department of Drug Discovery and Development, Harrison School of Pharmacy, Auburn University, Auburn, USA; 6 Department of Clinical Nutrition and Dietetics, Faculty of Nutrition Sciences and Food Technology, National Nutrition and Food Technology Research Institute, Shahid Beheshti University of Medical Science, Tehran, Iran

## Correction to: BMC Res Notes (2020) 13:221 https://doi.org/10.1186/s13104-020-05063-9

Following the publication of the original article [1] we were informed that the project No. 1398/61472 mentioned in the Acknowledgements section of the article is incorrect.

The correct project number is 20586.
